# Putting women in the centre of the global HIV response is key to achieving epidemic control!

**DOI:** 10.1002/jia2.25473

**Published:** 2020-03-06

**Authors:** Quarraisha Abdool Karim, Diane Havlir, Nittaya Phanuphak

**Affiliations:** ^1^ Centre for the AIDS Programme of Research in South Africa Nelson Mandela School of Medicine University of KwaZulu‐Natal Durban South Africa; ^2^ Department of Epidemiology Mailman School of Public Health Columbia University New York NY USA; ^3^ Department of Medicine University of California San Francisco CA USA; ^4^ PREVENTION Thai Red Cross AIDS Research Centre Bangkok Thailand

**Keywords:** women, HIV, prevention, treatment, transgender women, ageing

As we celebrate International Women's Day and the many successes that women have, and continue to achieve globally, we are also reminded of the many challenges that remain. HIV/AIDS continues to be one such challenge. In 2018, there were about 37 million people living with HIV, 900,000 AIDS‐related deaths and 1.4 million new HIV infections – a sharp reminder that HIV remains a major pandemic and that we have miles to go in sustaining our current treatment successes, strengthen our prevention efforts, and address continued human rights violations and research gaps before we can claim victory against HIV.

Globally, the distribution of HIV is uneven within and between regions. Vulnerable populations across the globe are also not homogeneous. Similarly, the vulnerability of women within and between countries and regions, by sexual orientation and across their life‐course differs. A nuanced and granular understanding is critical for the development of effective responses to reduce their vulnerability. A glimpse of this can be gleaned, for example, if we focus on adolescent girls and young women (AGYW; aged 15 to 24 years) – who in 2017 represented just 10% of the global population, yet accounted for 25% of all new HIV infections 1 – or transgender women who continue to face major barriers to access prevention and treatment services, or the millions of women who have benefitted from access to life‐saving antiretroviral treatment yet now face new challenges as they age. These issues are by no means exhaustive of the hardships facing women at risk of acquiring HIV or living with HIV, but we use the opportunity of this viewpoint to focus on these topics to share the prospects of and challenges to altering current global HIV trajectories for women and societies disproportionately impacted by HIV and to highlight the complexity and diversity of the issues facing women across their lifespan.

Sub‐Saharan Africa bears a disproportionate 70% of the global burden of HIV that is spread predominantly through sex with a concomitant epidemic in infants born to mothers living with HIV. A unique characteristic of this generalized epidemic is the high rate of HIV in AGYW, who are up to six times more likely to have HIV compared to their male peers. This early acquisition of HIV in young women is often from men who are five or more years older than them, and is central to the continued spread of HIV in this region. Four out of five of all infections occurring among adolescents in sub‐Saharan Africa are in girls aged 15 to 19 years. In South Africa, home to one in five of the world's people living with HIV, 1500 AGYW acquire HIV every week 1. Overall, AGYW are twice as likely to be living with HIV and experience higher rates of morbidity and mortality compared to their male peers.

Behavioural and biological factors such as age‐disparate sexual coupling between young women and older men 2, genital inflammation 3, 4 and vaginal dysbiosis 5 contribute to a young woman's vulnerability for acquiring HIV. These factors are difficult to mitigate against the backdrop of structural challenges of poverty 6, social incohesion due to migration and conflict, gender‐based violence 7 and gender‐power dynamics, including a woman's limited ability to negotiate safer sex with prevention technologies that depend on male co‐operation. When AGYW become pregnant, they are more likely to drop out of school. This limits their employment opportunities, leading to vicious cycles of dependency on older men, and increases their risk of having a repeat pregnancy within a year and of acquiring HIV if not already living with HIV 8. Preventing new infections in this group is therefore a high priority to alter the current epidemic trajectory in sub‐Saharan Africa and get it on the path of epidemic control. The PEPFAR‐funded DREAMS Project is an excellent example of an intervention targeting behavioural, biological and structural drivers of AGYW vulnerability, underscoring the importance of AGYW completing schooling, avoiding unplanned pregnancies and graduating from high school HIV‐free 9. These investments will strengthen social cohesion and realize the continent's full potential of youth as its demographic dividend.

Transgender women are among the most marginalized in society due to the general lack of legal gender recognition and criminalization of their gender identity. Stigma, discrimination and unavailability of transgender‐competent healthcare are key barriers for transgender women to address their basic health needs 10. In Thailand, for example, 48% of transgender women have never seen a doctor regarding the use of feminizing hormones and surgical procedures for their gender affirmation and 47% report having negative experiences when receiving health services due to their gender identity 11.

In Asia, between 2012 and 2018, HIV prevalence among transgender women ranged from 0.9% to 34% 12. Providing transgender‐led, integrated hormone and sexual health services has been a growing priority in the region over the past four years as a mechanism to enhance access to HIV‐related services. This effort has been led by the Tangerine Community Health Center in Bangkok, Thailand, where its services have significantly increased repeat HIV testing and PrEP uptake 13, 14. The model has recently been expanded to Hanoi and Ho Chi Minh City in Vietnam, and Manila in the Philippines 15.

Notwithstanding the expansion of this peer‐led intervention, only 5.8% of an estimated 9209 transgender women at high‐risk of HIV acquisition in Thailand have accessed PrEP since its adoption in 2015, 16 underscoring the need for more implementation research to scale‐up proven interventions, as well as research to identify innovative approaches to service delivery which are more friendly/tailored to transgender women.

Equally important to these advances in access to transgender‐competent health care is the need to strengthen legal protections and advocacy around human rights. On multiple levels, it is clear that transgender‐led research produce the most meaningful data. This research is being enhanced through increasingly collaborative trans‐cis effort in Asia as shown in examples above. Participatory trans‐cis research is crucial, and recent comments advocating the exclusion or minimization of cis‐gender researchers are not constructive 17, if we are going to accelerate a transgender research agenda.

Millions of women worldwide over the age of 50 are living with HIV – but their successes, challenges and future prospects are rarely heard. The majority of these women reside in sub‐Saharan Africa, but there are hundreds of thousands living in high‐, low‐ or middle‐income regions around the world. Congruent with expanded antiretroviral treatment access, their numbers will continue to increase over the next decade 18. We have been complacent about understanding the biomedical changes and societal barriers for these women; and research investment is lacking 19. Women, like men, are at increased risk for cardiac disease, malignancies and bone disease as they age 20; some conditions such as bone disease are amplified among peri‐ or post‐menopausal women. Knowledge of the consequences of the extraordinary weight gains associated with dolutegravir are as yet unknown, but differentially affect women 21. There are enormous unmet behavioural health needs for women throughout life, particularly associated with ageing, where depression and loneliness, coupled with cognitive decline, can lead to a downhill spiral and premature death 22.

The health and wellbeing of women ageing with HIV is also particularly affected by cultural norms that violate their fundamental human rights. Lack of property ownership and gender‐based violence can precipitate or exacerbate poverty, food security and access to care 23. Women in polygamous marriages may lose stature and resources as they age. Around the world, women over 50 who do not have HIV are often not considered “at risk” to acquire HIV, are tested less frequently than men, and are less likely to seek PrEP 24, 25. Yet, 22,000 women over 50 newly acquired HIV in southern and eastern Africa in 2018 alone 26.

Women across their lifespan fulfil many and multiple roles in society including being caretakers and educators in formal and informal settings, which largely goes unrecognized and is unrewarded. Moving towards the UN 2030 goal of ending AIDS as a public health threat calls for new and concerted efforts to:

1. ensure that innovation in prevention and treatment efforts include women, especially AGYW and pregnant women, from the outset to minimize data gaps and delays in access to effective interventions
2. prevent HIV in AGYW has to address the underlying gender‐power differences at the root cause of their vulnerability and therefore has to include testing and treatment targeting men and older women and gender sensitive interventions for adolescent boys including VMMC and comprehensive sexuality education.
3. strengthen transgender‐led effort to attain legal and human rights, as well as transgender‐competent care, including those related to HIV treatment and prevention
4. increase investments in research and care for all women of all ages to prevent or treat HIV
5. continued innovation for less user‐dependent women‐initiated HIV prevention technologies including vaccines are urgently needed to expand the array of safe and effective prevention options for women.

By ensuring that no women – from birth to death – experience barriers to access to the full range of HIV prevention and care services or violation of their basic human rights, all of society benefits.

## Competing interests

QAK and NP declared no competing interest. DH receives non‐financial support from Gilead Sciences.

## Authors’ contributions

DH, NP and QAK contributed equally to this viewpoint. QAK, DH and NP wrote the adolescent and young cisgender women, cisgender women who are ageing, and transgender women respectively. All authors reviewed critically and approved the final manuscript.
